# HBV Upregulates CtBP2 Expression via the X Gene

**DOI:** 10.1155/2018/6960573

**Published:** 2018-07-31

**Authors:** Xinghui Liu, Chengliang Zhu, Jie Li, Fengxia Xu, Gang Huang, Limin Xu, Binghong Zhang

**Affiliations:** ^1^Department of Clinical Laboratory, Shanghai Gongli Hospital, The Second Military Medical University, Pudong New Area, Shanghai 200135, China; ^2^Department of Clinical Laboratory, Renmin Hospital of Wuhan University, Wuhan, Hubei 430060, China; ^3^School Hospital, Huazhong University of Science and Technology, Wuhan, Hubei 430074, China; ^4^Department of Neonatology, Renmin Hospital of Wuhan University, Hubei 430074, China

## Abstract

**Background:**

Hepatitis B virus (HBV) infection causes acute and chronic liver diseases that can eventually develop into cirrhosis and hepatocellular carcinoma (HCC), but the carcinogenesis of HBV is not fully understood. Carboxyl-terminal-binding protein 2 (CtBP2) plays an important role in tumorigenesis and progression. The aim of this study was to investigate the effect of HBV on CtBP2 expression and to explore its mechanism.

**Methods:**

Real-time quantitative reverse transcription polymerase chain reaction (RT-qPCR) and western blotting were used to evaluate the CtBP2 mRNA and protein expression levels in tissues and cells. The HBV infectious clone pHBV1.3 and plasmids expressing a single gene of the HBV genome were cotransfected with the CtBP2 gene promoter pGL3-CtBP2 into the human hepatoma cell line HepG2, and luciferase activity was determined using a luminometer.

**Results:**

CtBP2 expression was higher in HBV-related HCC tissues than in paracancerous tissues. CtBP2 expression was higher in HepG2.2.15 cells integrated with the HBV genome than in HepG2 cells. pHBV1.3 upregulated CtBP2 mRNA and protein expression. The HBV X gene significantly activated CtBP2 gene promoter activity, and CtBP2 mRNA and protein expression were upregulated by the HBV X gene. This activation effect was enhanced by the increase in the dose of the X gene, showing metrological dependence.

**Conclusion:**

HBV may be involved in the occurrence and development of HCC by upregulating CtBP2 expression.

## 1. Introduction

Primary hepatocellular carcinoma (HCC) is highly malignant. Currently, hepatitis B virus (HBV) is commonly recognized as one of the main causes of HCC. More than 2 billion people are infected with HBV worldwide, with an estimated 320 thousand deaths occurring every year, of which approximately 30-50% are due to HCC. Clinical studies have shown that the risk of HCC is 200 times higher in individuals with chronic HBV infections than in the general people without HBV infections [1–3]. HBV contains four open reading frames (S, C, P, and X). Among them, the X gene is closely related to carcinogenicity. This gene encodes the X protein, which has 154 amino acid residues and a mean molecular weight of 17 kDa. The X protein plays an important role in the occurrence and development of HCC and participates in the proliferation and transformation of liver cells [4, 5]. Carboxyl-terminal-binding protein 2 (CtBP2) is an important member of its family and is located on chromosome 21q21.3 [6]. Many recent studies have found that CtBP2 expression is closely related to tumors [7–10]. In our previous study, a gene microarray was used to screen HBV-related differentially expressed genes in HCC tissues and paracancerous tissues [11]. The results showed that CtBP2 expression was higher in the HCC tissues. To date, no relevant research on HBV and CtBP2 has been reported. This study investigated the regulation of CtBP2 by HBV and the underlying molecular mechanism.

## 2. Materials and Methods

### 2.1. Clinical Specimens and Tissues

Thirty-five cases of primary HCC specimens with a history of HBV infection were collected after surgical resection in the Department of Hepatobiliary Surgery, Zhongnan Hospital. Paracancerous liver tissues were also collected from the above HCC patients as controls. The fresh specimens were placed in liquid nitrogen for cryopreservation immediately after surgical resection. This study was approved by the Ethics Committee, and all subjects signed the informed consent form.

### 2.2. Cell Culture and Transfection

The human hepatoma cell lines HepG2 and HepG2.2.15 were cultured in high-glucose Dulbecco's modified Eagle's medium (DMEM) containing 10% fetal bovine serum and 1% streptomycin and were incubated in a 37°C and 5% CO_2_ incubator. When the cell growth reached the logarithmic phase, the HepG2 cells were seeded into 24- or 6-well plates. When the cell confluence reached 80%, the operations were conducted following the kit instructions. First, 0.6 *μ*g of the eukaryotic expression plasmid containing the HBV genome (PCMV-S, pCMV-E, pCMV-C, pCMV-X, and pCMV-P) and 2 *μ*L of the Lipofectamine 2000 transfection reagent were diluted in 30 *μ*L of DMEM or 2 *μ*g of pCMV-X plasmid DNA and 4 *μ*L of the Lipofectamine 2000 transfection reagent were diluted in 100 *μ*L of DMEM. The transfection solutions were incubated at room temperature for 20 min and then added to the culture medium in the 24- or 6-well plates. The cells were cultured in the CO_2_ incubator.

### 2.3. Real-Time Quantitative Reverse Transcription Polymerase Chain Reaction (RT-qPCR)

The cells were harvested, and 1 mL of the TRIzol reagent were added to extract the total RNA. cDNA was synthesized from 1 *μ*g of total RNA by reverse transcription. After the cDNA was diluted 10-fold, 1 *μ*L of cDNA was added to each PCR reaction. The CtBP2 gene was quantitatively detected using the SYBR® GreenER qPCR kit. The primer sequences were as follows: CtBP2 (sense): 5′-CGT TCT CAG AGC TGG GAT GC-3′ and CtBP2 (antisense): 5′-TCT GCT GTG CCA TAC GTC AG-3′. The GAPDH gene was used as the internal reference [12].

### 2.4. Determination of Luciferase Activity

The cells were harvested and lysed with cell lysis buffer. After mixing 10 *μ*L of cell lysate and 100 *μ*L of luciferase substrate, the luciferase activity was measured with a luminometer.

### 2.5. Western Blotting

Total protein was extracted from the tissues and cells and boiled for 5 min in loading buffer. The sodium dodecyl sulfate–polyacrylamide gel electrophoresis (SDS-PAGE) stacking gel and 10% separation gel were prepared. A total of 30 *μ*g of protein was loaded onto each well. The electrophoresis was performed at 60 V for the stacking gel and at 110 V for the separation gel. The separated proteins were transferred onto the membrane at 100 V for 70 min. After blocking with 8% skimmed milk for 2 h, the membrane was incubated with the primary antibody overnight at 4°C, followed by 3 washes with phosphate-buffered saline with Tween-20 (PBST) and then incubated with the secondary antibody at room temperature for 2 h. After washing with PBST again, the appropriate amount of electrochemiluminescence (ECL) solution was added onto the membrane in a dark room, and the membrane was placed on a protein gel imaging system for imaging.

### 2.6. Statistical Analysis

All data are represented as the mean ± standard deviation. SPSS 20.0 statistical analysis software was used for data processing. The means were compared using a t-test. Additionally, correlation between HBx and CtBP2 was analyzed by Pearson correlation coefficients; differences with p < 0.05 were considered significant.

## 3. Results

### 3.1. CtBP2 Expression Was Higher in the HBV-Related HCC Tissues Than in the Paracancerous Tissues

**Figure 1:**
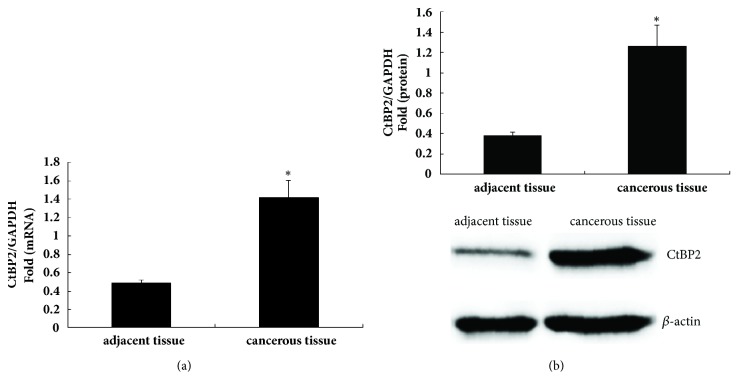
CtBP2 expression in HBV-related HCC tissues. (a) Total RNA was extracted using TRIzol from thirty-five pairs of HBV-related HCC tissues and paired paracancerous liver tissues, and CtBP2 mRNA expression was detected by RT-qPCR. (b) Total protein was extracted from thirty-five pairs of HBV-related HCC tissues and paired paracancerous liver tissues, and CtBP2 protein expression was detected by western blotting.*∗*p < 0.05.

The CtBP2 expression levels in the thirty-five pairs of paired HBV-related HCC tissues and paracancerous liver tissues from each of patients were measured by RT-qPCR and western blotting. The CtBP2 mRNA and protein expression levels were higher in the HCC tissues than in the paracancerous liver tissues (Figures 1(a) and 1(b)).

### 3.2. RNA and Protein Expression Were Upregulated by HBV in HepG2 Cells

**Figure 2:**
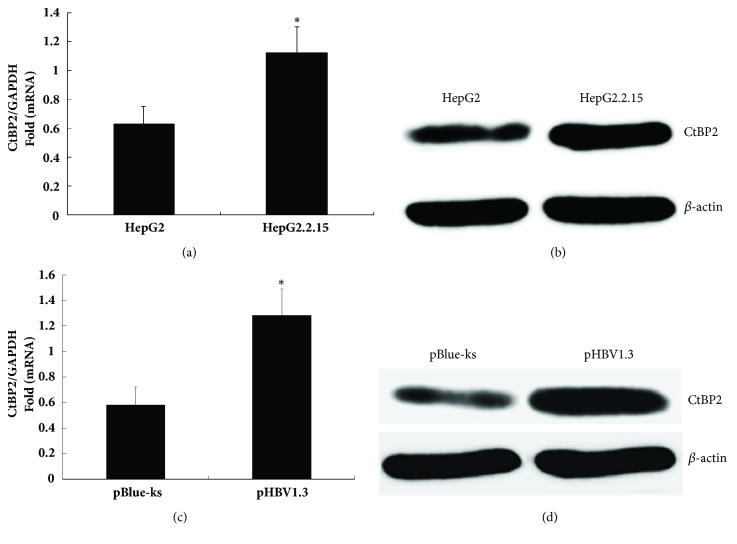
Effect of HBV on CtBP2 expression in HepG2 cells. (a) TRIzol was used to extract the total RNA from HepG2.2.15 cells integrated with the HBV genome and the control HepG2 cells, and mRNA expression was detected by RT-qPCR. (b) Total protein was extracted from the HepG2.2.15 cells and the control HepG2 cells, and protein expression was detected using western blotting; (c) HepG2 cells were transfected with 0.6 *μ*g of HBV1.3 and the control plasmid pBlue-ks, and CtBP2 mRNA expression was detected by RT-qPCR after 48 h; (d) HepG2 cells were transfected with 0.6 *μ*g of HBV1.3 and the control plasmid pBlue-ks, and CtBP2 protein expression was detected by western blotting after 48 h.

Using RT-qPCR and western blotting, the CtBP2 expression levels were measured in HepG2.2.15 cells integrated with the whole HBV genome and in control HepG2 cells. The CtBP2 mRNA and protein expression levels were higher in the HepG2.2.15 cells than in the HepG2 cells (Figures 2(a) and 2(b)), which was consistent with the gene microarray screening results.

The HBV infectious clone pHBV1.3 was transfected into HepG2 cells, and transfection with the pBlue-ks vector was used as the blank control. After 48 hours, the mRNA and total proteins were extracted from the cell lysate, and CtBP2 expression was detected by RT-PCR and western blotting. The CtBP2 mRNA and protein expression levels were higher after transfection with pHBV1.3 than after transfection with the control plasmid pBlue-ks (Figures 2(c) and 2(d)), indicating that HBV upregulated the CtBP2 mRNA and protein expression levels.

### 3.3. HBV X Gene Expression Activated the CtBP2 Promoter

**Figure 3:**
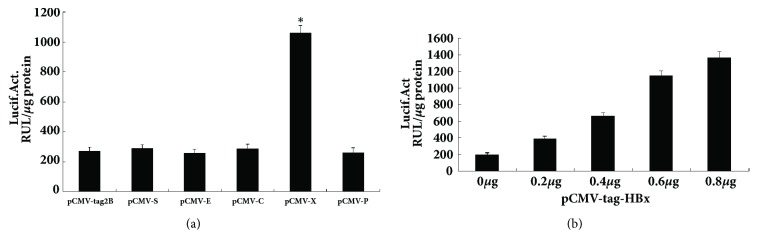
Effect of the HBV X gene on CtBP2 promoter activity. (a) Luciferase activity was measured 48 h after cotransfection of 0.6 *μ*g of the eukaryotic expression plasmid containing each gene of the HBV genome (pCMV-S, pCMV-E, pCMV-C, pCMV-X, and pCMV-P) and 0.2 *μ*g of the CtBP2 gene promoter pGL3-CtBP2 into HepG2 cells using pCMV-tag2B as the blank control. Each experiment was repeated three times. (b) Different doses of pCMV-X (0 *μ*g, 0.2 *μ*g, 0.4 *μ*g, 0.6 *μ*g, and 0.8 *μ*g) and pGL3-CtBP2 were cotransfected into HepG2 cells, and the luciferase activity was detected after 48 h. *∗*p < 0.05.

Each of the eukaryotic expression plasmids containing all of the genes of the HBV genome (pCMV-S, pCMV-E, pCMV-C, pCMV-X, and pCMV-P) and the pBP3-CtBP2 promoter were cotransfected into HepG2 cells with pCMV-tag2B as the blank control. Activation of the CtBP2 gene promoter by these proteins was detected using a luciferase activity assay. The luciferase activities detected after transfection with pCMV-S, pCMV-E, pCMV-C, pCMV-X, pCMV-P, and pCMV-tag2B were 268.42 ± 28.16 RUL/*μ*g protein, 289.33 ± 24.45 RUL/*μ*g protein, 256.61 ±27.39 RUL/*μ*g protein, 285.42 ± 31.74  RUL/*μ*g protein, 1058.65 ± 52.39 RUL/*μ*g protein, and 260.53 ± 33.58  RUL/*μ*g protein, respectively. The HBV X gene had a significant activation effect on the CtBP2 gene promoter, whereas the other HBV genes showed no significant activation effects (Figure 3(a)).

To investigate the activation of the CtBP2 promoter by the HBV X gene, the eukaryotic expression plasmid for the X gene (pCMV-X) was cotransfected into HepG2 cells at different doses (0 *μ*g, 0.2 *μ*g, 0.4 *μ*g, 0.6 *μ*g, and 0.8 *μ*g) with pGL3-CtBP2. The effect of the different doses of the X gene on pG3-CtBP2 activation was detected using the luciferase activity assay. The luciferase activities after transfection of the X gene were 196.35 ± 24.22 RUL/*μ*g protein, 385.68 ± 28.47 RUL/*μ*g protein, 655.15 ± 39.34 RUL/*μ*g protein, 11143.48 ± 63.18 RUL/*μ*g protein, and 1365.79 ± 69.42 RUL/*μ*g protein for the doses of 0 *μ*g, 0.2 *μ*g, 0.4 *μ*g, 0.6 *μ*g, and 0.8 *μ*g, respectively, demonstrating that the CtBP2 promoter activity increased with the increase in X protein expression (Figure 3(b)). Thus, the HBV X gene can specifically activate the CtBP2 gene promoter.

### 3.4. CtBP2 mRNA and Protein Expression Were Upregulated by the HBV X Gene

**Figure 4:**
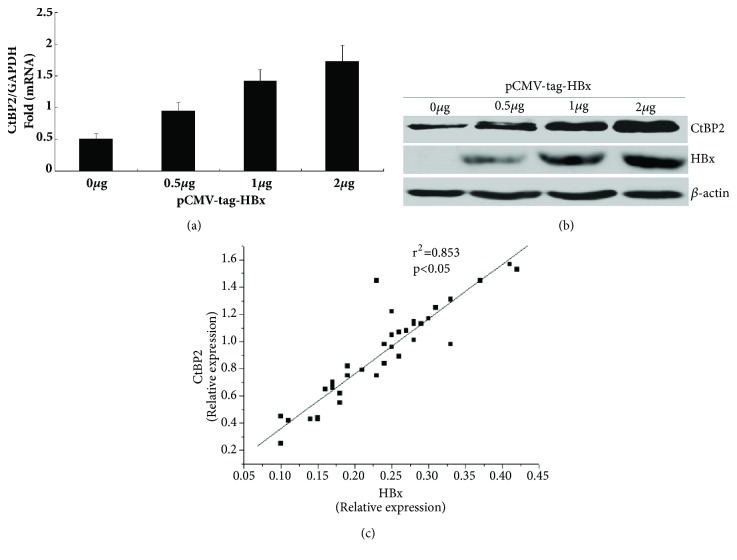
Effect of the HBV X gene on CtBP2 mRNA and protein expression. (a) CtBP2 mRNA expression was detected by RT-qPCR 48 h after transfection of different doses of the X gene eukaryotic expression plasmid pCMV-X (0 *μ*g, 0.5 *μ*g, 1 *μ*g, and 2 *μ*g) into HepG2 cells. (b) CtBP2 protein expression was detected by western blotting 48 h after transfection of different doses of the X gene eukaryotic expression plasmid pCMV-X (0 *μ*g, 0.5 *μ*g, 1 *μ*g, and 2 *μ*g) into HepG2 cells. (c) Correlation between HBx and CtBP2 expression in HBV-related HCC tissues.

To investigate the effect of the HBV X gene on CtBP2 expression at the transcription and translation levels, different doses of the X gene eukaryotic expression plasmid pCMV-X (0 *μ*g, 0.5 *μ*g, 1 *μ*g, and 2 *μ*g) were transfected into HepG2 cells for 48 h, and CtBP2 mRNA and protein expression were detected by qPCR and western blotting. The CtBP2 mRNA and protein expression levels increased in a dose-dependent manner with the increase in the X gene concentration (Figures 4(a) and 4(b)), which was consistent with the luciferase activity assay results. Furthermore, positive correlation between HBx and CtBP2 expression was found in HCC tissues using the Pearson correlation analysis (Figure 4(c)). Thus, the HBV X gene upregulated CtBP2 expression at the transcription and translation levels.

## 4. Discussion

Recently, protein differential screening, protein microarray, and protein mass spectrometry technologies have been widely applied, and an increasing number of HBV pathogenic factors have been discovered. To further explore the pathogenesis and carcinogenesis of HBV, in our previous study we used gene microarray technology to screen differentially expressed genes in HepG2 and HepG2.2.15 cells as well as HBV-related HCC tissues and paracancerous tissues and found that CtBP2 was highly expressed in the HepG2.2.15 cells and HCC tissues (data not shown). Because certain false positives are present in microarray screening results, the results were verified using RT-PCR and western blotting. The results showed that the CtBP2 expression levels were higher in the three pairs of HCC tissues than in the paracancerous tissues and that CtBP2 expression was higher in the HepG2.2.15 cells than in the HepG2 cells, which was consistent with the gene microarray screening results. Then, the HBV infectious clone pHBV1.3 was transfected into HepG2 cells; the results showed that HBV upregulated CtBP2 mRNA and protein expression in the HepG2 cells.

To explore the regulatory mechanism underlying the effect of HBV on CtBP2, the CtBP2 gene promoter was cloned into the pGL3-basic vector containing a luciferase reporter gene, and regulation of the CtBP2 promoter by HBV was detected using the luciferase reporter gene system. All genes in the HBV genome were cotransfected with the CtBP2 gene promoter into HepG2 cells. By measuring the luciferase activity, we found that the HBV X gene activated the CtBP2 gene promoter and that this activation effect was dose-dependent, indicating that CtBP2 expression was upregulated by the X gene at the promoter level. Furthermore, CtBP2 mRNA and protein expression were detected by RT-qPCR and western blotting. The results showed that the HBV X gene upregulated CtBP2 mRNA and protein expression in a dose-dependent manner, indicating that HBV activated CtBP2 expression via the X gene.

HBV can regulate the abnormal expression of many cellular proteins in an HBV-infected organism, and these proteins are involved in tumorigenesis [11–16]. The HBx gene has the smallest open reading frame in the HBV genome. As a transactivating factor, HBx can bind to a variety of factors related to transcription and gene regulation and widely activate the promoters of the virus and cells. Thus, HBx is involved in regulation of host gene expression [17–21].

The results of the study showed that CtBP2 played an important role in the development and progression of tumors. For example, in colorectal cancer, the interaction of CtBP2 with TCF-4 can activate its transcription activity, leading to activation of the downstream target gene *β*-catenin and resulting in cell migration [22]. Overexpression of CtBP2 in lung cancer cells can induce the dephosphorylation of its target gene PTEN and enhance the cell proliferation ability [22, 23]. Moreover, CtBP2 can also be used as an independent prognostic marker after hepatectomy [24]. HBV is well recognized as one of the most important causes of HCC, which can lead to the occurrence of liver cancer [25, 26]. Therefore, HBV may upregulate CtBP2 expression via its HBx gene and thus promote the occurrence and development of HCC.

## 5. Conclusions

This study investigated the regulation of CtBP2 expression by HBV and explored its regulatory mechanism at the molecular level. These results laid a foundation for elucidating the pathogenesis and carcinogenesis of HBV. However, further investigation is needed to identify the specific signaling pathways involved in the regulation of CtBP2 expression by HBx and the role of CtBP2 in HCC.

## Data Availability

The dataset supporting the conclusions of this article is included within the article.
